# Efficacy and safety of acupuncture for chronic dizziness: study protocol for a randomized controlled trial

**DOI:** 10.1186/1745-6215-14-429

**Published:** 2013-12-13

**Authors:** Zhe Xue, Cun-Zhi Liu, Guang-Xia Shi, Yan Liu, Zhao-Xin Li, Zhen-Hua Zhang, Lin-Peng Wang

**Affiliations:** 1 Acupuncture and Moxibustion Department, Beijing Hospital of Traditional Chinese Medicine affiliated to Capital Medical University, 23 Meishuguanhou Street, Dongcheng District, Beijing 100010, China; 2 Shandong University of Traditional Chinese Medicine, Jingshi Street, Lixia District, Jinan 250014, China

**Keywords:** Acupuncture, chronic dizziness, randomized controlled trial

## Abstract

**Background:**

Dizziness is one of the most challenging symptoms in medicine. No medication for dizziness in current use has well-established curative or prophylactic value or is suitable for long-term palliative use. Unconventional remedies, such as acupuncture, should be considered and scientifically evaluated. However, there has been relatively little evidence in randomized controlled clinical trials on acupuncture to treat chronic dizziness. The aim of our study is to evaluate the efficacy and safety of acupuncture in patients with dizziness.

**Methods/Design:**

This trial is a randomized, single-blind, controlled study. A total of 80 participants will be randomly assigned to two treatment groups receiving acupuncture and sham acupuncture treatment, respectively, for 4 weeks. The primary outcome measures are the Dizziness Handicap Inventory (DHI) and the Vertigo Symptom Scale (VSS). Treatment will be conducted over a period of 4 weeks, at a frequency of two sessions per week. The assessment is at baseline (before treatment initiation), 4 weeks after the first acupuncture session, and 8 weeks after the first acupuncture session.

**Discussion:**

The results from this study will provide clinical evidence on the efficacy and safety of acupuncture in patients with chronic dizziness.

**Trial registration:**

International Standard Randomized Controlled Trial Number Register: ISRCTN52695239

## Background

Dizziness is one of the most challenging symptoms in medicine, and the prevalence has been reported to be between 11.1% and 28.9% [1]. It is associated with functional disability, and 10 to 20% of sufferers fall because of their symptoms [2]. Although spontaneous remission is possible, many patients experience delays in vestibular compensation, which lead to chronic symptoms that interfere with everyday functional tasks [3]. Over 66% of dizzy patients also experience psychological distress, often resulting in a vicious cycle of fear, avoidance of symptom-provoking movements, and increased handicap, retarding recovery even further [4,5].

Dizziness can be produced by peripheral vestibular disorders, central nervous system disorders, or combined lesions, as well as other conditions [2]. Treatment typically consists of reassurance and antivertiginous and antiemetic drugs to relieve symptoms [6-9]. However, several reviews of the management of dizziness have concluded that no medication in current use has well-established curative or prophylactic value or is suitable for long-term palliative use [10]. Unconventional remedies, such as acupuncture, should be considered and scientifically evaluated [11]. Acupuncture, which is one of the main treatment modalities of traditional Chinese medicine, has been used for both the prevention and treatment of chronic dizziness for over three thousand years. The rapid development of acupuncture both within and outside China over the last few decades has itself led to great innovations in practice. Many studies, including clinical reports and systematic reviews, have investigated the benefits and success of acupuncture in relieving symptoms for various chronic diseases [12]. However, there has been relatively little evidence in randomized controlled clinical trials on acupuncture to treat chronic dizziness.

The aim of our study is to evaluate the efficacy and safety of acupuncture in patients with dizziness.

## Methods/Design

This is a randomized, controlled prospective study. We will perform the study according to common guidelines for clinical trials (Declaration of Helsinki, International Conference on Harmonisationand WHO Good Clinical Practice standards, including certification by an external audit). The trial protocol has been approved by the Research Ethical Committee of Beijing Hospital of Traditional Chinese Medicine Affiliated to Capital Medical University (ref: 201316). This trial was registered with ISRCTN at Current Controlled Trials (ISRCTN52695239).

### Population

**Figure 1:**
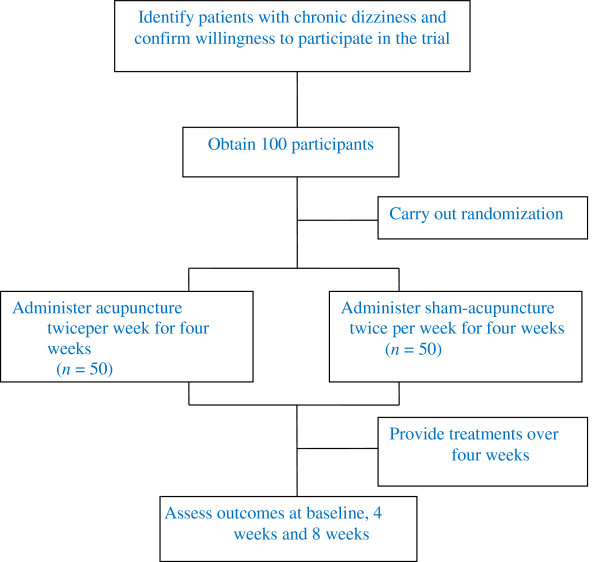
Study sequence.

Patients will be recruited in the acupuncture clinic at Beijing Hospital of Traditional Chinese Medicine, affiliated to Capital Medical University, with a target sample size of 100 subjects. Our trial will be promoted by a poster at this hospital. All candidates will go through a standardized interview process and receive more information about the study and the treatments. Patients will be informed in a manner suggesting that two different types of acupuncture treatment will be compared. One type will be traditional acupuncture and the other will be a new type of acupuncture, and the efficacy of both types is uncertain. Terms such as ‘placebo’ or ‘sham’ will not be mentioned. The participants’ written consents will be obtained. The purpose and procedures, and potential risks and benefits of the study will also be explained thoroughly to the participants. Participants will be able to withdraw from the study at any time without consequence. The trial will be executed from February 2013 to December 2015. All recruit procedures will be recorded in a log file (Figure 1).

### Inclusion criteria

Participants meeting the following criteria will be included:

1. Vertigo of unknown cause;

2. Dizziness of unknown cause;

3. Ménière’s disease;

4. Psychogenic dizziness;

5. Age 18 to 75 years, either sex.

### Exclusion criteria

Participants meeting one or more of the following criteria will be excluded:

1. Labyrinthitis;

2. Benign positional vertigo;

3. Vestibular neuronitis;

4. Duration of dizziness less than 2 months in the past 2 years;

5. Serious comorbid conditions (for example, life-threatening condition or progressive central disorder);

6. Patients who cannot communicate reliably with the investigator or who are not likely to cope with the requirements of the trial;

7. Patients receiving or received acupuncture treatment in past 3 month.

### Interventions

Patients who meet the inclusion criteria and none of the exclusion criteria are randomized to one of two treatment groups. Group A will receive acupuncture in the Baihui, Yintang, Taiyang, Tinggong, Wangu, Fengchi, Hegu, Fenglong, and Taichong acupuncture points (Table 1). Single-use, sterile, 40 mm × 0.25 mm or 25 mm × 0.25 mm (depending on the area to be treated), silver-handled needles (HanYi disposable acupuncture needle, made in Tianjin HuaHong Medical Co., Ltd., Tianjin, China) without guide tubes are used. Needles are correctly inserted and manually stimulated until the ‘De Qi’ sensation is elicited. The needles will stay in place for 30 min and will be manually stimulated every 10 min. Only needle acupuncture will be allowed: other forms of acupuncture treatment (for example, laser acupuncture, electro-acupuncture, or moxibustion) will not be permitted.

**Table 1:** Acupoints and needling procedure for real acupoint in this trial

**Acupoints**	**Meridian**	**Puncturing angle**	**Depth (cun)**	**Manipulation**	**Twisting angle (degrees)**	**Frequency (per minutes)**
Baihui (GV20)	Du meridian	Transversely (unilateral)	0.5 to 0.8	Twirling, reinforcing, manipulation	<90	120
Yintang (EX-HN3)	Extra nervepoint	Transversely (unilateral)	0.3 to 0.5	Moderatereinforcing, reducing, twisting, manipulation	90 to 180	60 to 120
Taiyang (EX-HN5)	Extra nervepoint	Perpendicularly (bilateral)	0.3 to 0.5	Moderatereinforcing, reducing, twisting, manipulation	90 to 180	60 to 120
Tinggong (SI19)	Small intestine, meridian	Perpendicularly (bilateral)	1 to 1.5	Twirling, reinforcing, manipulation	<90	>120
Wangu (GB12)	Gallbladdermeridian	Transversely (bilateral)	0.5 to 0.8	Twirlingreinforcing, manipulation	<90	>120
Fengchi (GB20)	Gallbladdermeridian	Obliquelydownwardto the nose (bilateral)	0.8 to 1.2	Twirling, reinforcing, manipulation	<90	>120
Hegu (LI4)	Large intestinemeridian	Perpendicularly (bilateral)	0.5 to 1	Moderatereinforcing, manipulation	90 to 180	60 to 120
Fenglong (ST40)	Stomachmeridian	Perpendicularly (bilateral)	1 to 1.5	Twirling, reducing, twisting, manipulation	>180	<60
Taichong (LR3)	Livermeridian	Perpendicularly (bilateral)	0.5 to 0.8	Twirling, reducing, manipulation	>180	<60

Group B will receive sham acupuncture (shallow needle insertion that does not penetrate below the skin) at non-acupoints. The locations of non-acupoints have been defined in the previous literature [13]. The same type of needle will be used as group A, but the insertion is superficial and physicians are not instructed to achieve ‘De Qi’.

Treatment will be conducted over a period of 4 weeks, at a frequency of two sessions per week. No additional treatment will be allowed.

### Education of acupuncturists

Acupuncturists will have an acupuncture license from the Chinese medicine practitioner license from the Ministry of Health of the People’s Republic of China and take an educational course to ensure their strict adherence to the study protocol and familiarity with the administering study.

### Randomization and blinding

A research coordinator will screen and enroll participants at the clinic. After participants have completed a baseline evaluation, another research coordinator who is uninvolved with data collection randomly assigns them to one of two treatment groups by using a computer-generated, blocked random-allocation sequence. The random list is generated with SAS software (SAS Institute, Inc., Cary, NC, USA). This research coordinator informs the acupuncturist of the treatment assignment. The allocation of the randomization sequence will be concealed centrally by telephone.

The patients, data collection staff, and data analysts are blinded during the study period. The acupuncturist is not blinded to the treatment to be delivered because acupuncture manipulation makes this impossible. During the intervention, the acupuncturist and the personnel who collect data will be segregated immediately after the treatment has started and will be instructed not to exchange information with each other.

### Primary outcome measures

#### Dizziness Handicap Inventory

The Dizziness Handicap Inventory (DHI) is a validated, self-reported questionnaire, which is widely used to evaluate the functional, emotional, and physical impact of dizziness on patients’ daily life. It consists of 25 questions about daily problems associated with dizziness, and each question is given a score of 0, 2, or 4. A score of 0 means that the condition described in the question never happens, 2 means it sometimes happens, and 4 means it always happens. Followinga study by Whitney *et al.*[14], the DHI scores are graded as mild (0 to 30 points), moderate (31 to 60 points), and severe (61 to 100 points) [15,16].

#### Vertigo Symptom Scale, Short Form

The Vertigo Symptom Scale (VSS) is used as a measure of dizziness symptoms. The VSS assesses how frequently participants have experienced 15 various forms of dizziness during the previous month. It is rated on a scale ranging from 0 to 4 and has a maximum score of 60. Higher scores indicate more vertigo symptoms [17,18].

The DHI and VSS are assessed at baseline (before treatment initiation), 4 weeks after the first acupuncture session, and 8 weeks after the first acupuncture session.

### Secondary outcome measures

#### Hospital Anxiety and Depression Scale

The Hospital Anxiety and Depression Scale (HAD) independently assesses nonsomatic symptoms of anxietyand depression. Composite scores range between 0 and 21. Scores between 8 and 10 are borderline values and those above 10 indicate clinical depression or anxiety [4].

#### Short Form-36 Quality-of-Life Questionnaire

The Short Form-36 Quality-of-Life Questionnaire (SF-36) is a widely used tool for evaluating a person’s health perception in daily life. It consists of 36 questions, which are grouped into eight health categories as follows: (1) limitations in physical function because of health problems; (2) limitations in social function because of physical or emotional problems; (3) limitations in usual role activities because of physical health problems; (4) bodily pain; (5) general mental health (psychological distress and well-being); (6) limitations in usual role activities because of emotional problems; (7) vitality (energy and fatigue); and (8) general health perceptions [15].

#### Treatment Credibility Scale

Perceived credibility of acupuncture will be evaluated by the Treatment Credibility Scale after a 4-week acupuncture session. This is a five-item questionnaire ranging from 1 (not at all) to 5 (very confident): items are averaged to provide a single treatment credibility score, with high scores reflecting high treatment credibility [13].

To evaluate the adequacy of blinding, we will ask participants to rate how certain they are that they have received traditional acupuncture or the new method of acupuncture on a seven-point scale (1 very sure, 7 very uncertain) after 4 weeks of treatment [13].

The HAD and SF-36 will be assessed at baseline (before treatment initiation), 4 weeks after the first acupuncture, and 8 weeks after the first acupuncture. The Treatment Credibility Scale and the adequacy of blinding will only be evaluated after the acupuncture session on the fourth week.

### Safety

Participants should report any adverse events they experience, including discomfort or bruising at the sites of needle insertion, nausea, or feeling faint after each treatment.

### Sample size

We have performed sample size calculations for chronic dizziness according to other studies on the same disease using the same primary outcome measure because no such acupuncture research has been reported [19-21]. The estimated sample size was calculated considering previous DHI results. Calculations are performed using 80% power and a (two-sided) 5% significance level. Estimations indicated 263 individuals for the DHI assessments (effect size = 0.41; standard deviation = 0.7), where effect size is the mean difference expected between the two groups and standard deviation is for the population. We plan to enroll a total of 100 participants with 50 in each group, allowing for a 15% withdrawal rate.

### Data analysis

Descriptive statistics will be used to describe demographic and baseline characteristics of study participants. Repeated measuresanalysis of variance will be used to test the between-group differences. The accepted level of significance for all analyses will be *P* < 0.05. Data analysis will be conducted by statisticians who are independent of the research team. Analysis will be conducted using SPSS software (SPSS 12.0 KO for Windows ©).

## Discussion

Our study is, to date, the first randomized controlled clinical trial on the efficacy of acupuncture in patients with chronic dizziness.

The word dizziness is used to mean various sensations of body orientation and position that are frequently difficult for patients to describe. Dizziness is difficult to define, impossible to measure, and a challenge to diagnose [22]. Most patients with chronic symptoms are not relieved by medical treatment [23]. Chronic dizziness is a major public health concern. There are millions of patients with chronic dizziness. However, neither the mechanism nor safe and effective treatment methods have been clarified. Any treatment method that reduces chronic dizziness would be an important intervention. Our study will indicate that, despite the difficulty of diagnosis, practitioners can identify patients who can safely benefit from acupuncture. A number of patients with dizziness would prefer acupuncture over pharmacotherapy when our study demonstrates the effectiveness and safety of acupuncture to treat chronic dizziness. This will be especially relevant for patients who do not respond to medical therapy or who experience adverse side effects to drug therapy.

In our study, the acceptability of acupuncture will be reported by patients. Our findings provide insight into a key factor related to effectiveness, namely acceptability of treatment. Acceptability is an important factor because it will impact on the take-up of the treatment offered and the compliance or willingness to see the course of treatment through [24]. We will report on patients’ experiences of acceptability, whether the outcomes from acupuncture are beneficial or not.

However, our study has several limitations. One limitation concerns the fact that we will recruit patients with various types of chronic dizziness, although all the patients referred to acupuncture department will be eligible for the study. However, effectiveness of acupuncture for different types of chronic dizziness is not evaluated. Another limitation is that the therapist is not blinded in the present trial. A blinded study of acupuncture would be challenging to conduct because it is almost impossible to blind acupuncturists to the treatments they are delivering. Therefore, a bias due to unblinding cannot be ruled out.

The results of this study will provide a new evidence-based treatment option for patients suffering chronic dizziness.

## Trial status

The trial is currently in the recruitment phase.

## Abbreviations

DHI: Dizziness handicap inventory; HAD: Hospital anxiety and depression scale; SF-36: Short form-36 quality-of-life questionnaire; VSS: Vertigo symptom scale.

## Competing interests

The authors declare that they have no competing interests.

## Authors’ contributions

CZL conceived the study and prepared the initial protocol. ZX drafted the manuscript and participated in the study design. GXS participated in the development of the acupuncture point protocol. YL was in charge of coordination and directed implementation. ZXL and ZHZ helped to develop the study analysis. LPW made amendments and participated in designing the trial protocol. All authors read and approved the final manuscript.
